# Supplementary material for: A ketone/alcohol polymer for cycle of electrolytic hydrogen-fixing with water and releasing under mild conditions
Source: Nat Commun. 2016 Sep 30;7:13032. doi: 10.1038/ncomms13032 (PMC5427515; doi:10.1038/ncomms13032)
Supplement: Supplementary Information — Supplementary Figures 1-9, Supplementary Table 1 and Supplementary Methods [file ncomms13032-s1.pdf]

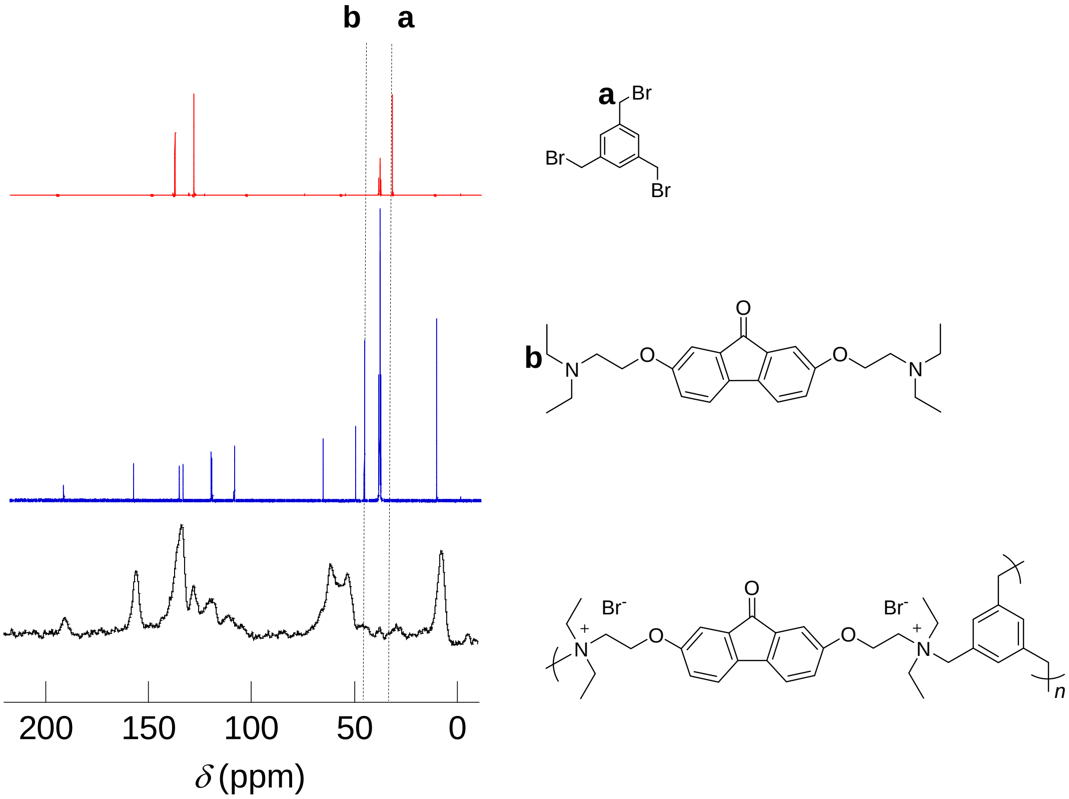

Supplementary Figure 1 | ^{13}C NMR spectra of the fluorenone polymer. Solid-state ^{13}C NMR spectrum of the fluorenone polymer and solution-state spectra of the reactants: 1,3,5-tris(bromomethyl)benzene and 2,7-bis[2-(diethylamino)ethoxy]-9-fluorenone in DMSO-d_6. Shifts of the methylene carbons of reactants in the bottom spectrum supported the polymerization progress.

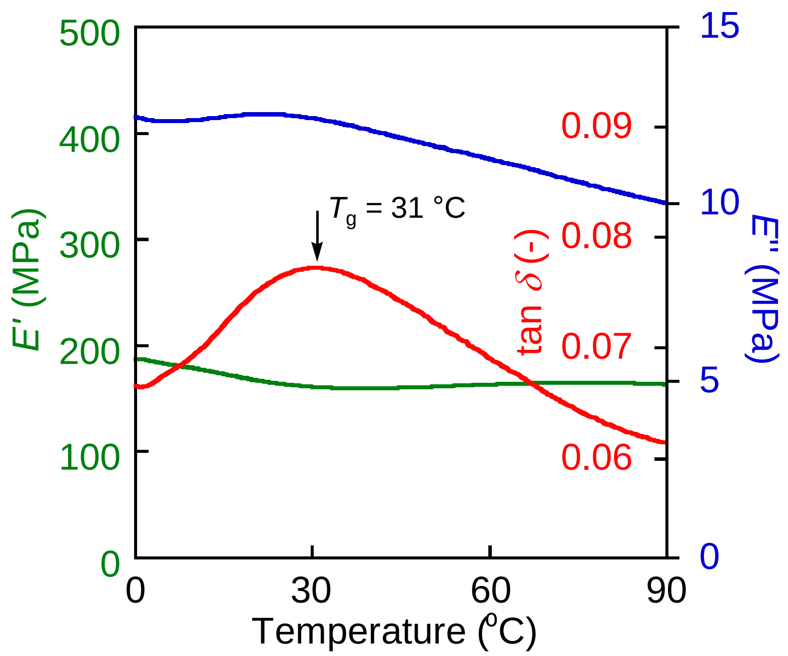

Supplementary Figure 2 | Dynamic mechanical measurement of the fluorenone polymer. Storage modulus (*E'*), loss modulus (*E"*), and tangent delta were analyzed by the dynamic mechanical measurement.

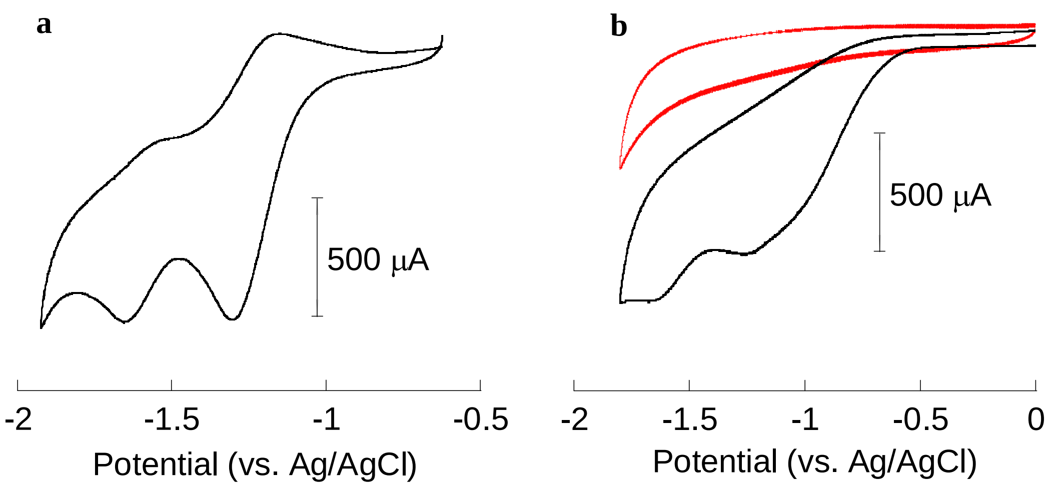

Supplementary Figure 3 | Cyclic voltammograms of the fluorenone polymer-coated carbon. a, The cyclic voltammogram of fluorenone polymer-coated carbon scanned at 50 mV s^{-1} in 0.1 M $(C_4H_9)_4NPF_6$ AN solution. **b,** The cyclic voltammograms of the fluorenone polymer-coated carbon scanned at 50 mV s^{-1} in 0.1 M $(C_4H_9)_4NPF_6$ AN solution containing a drop of water (black line), and after multiple potential cycles (red line).

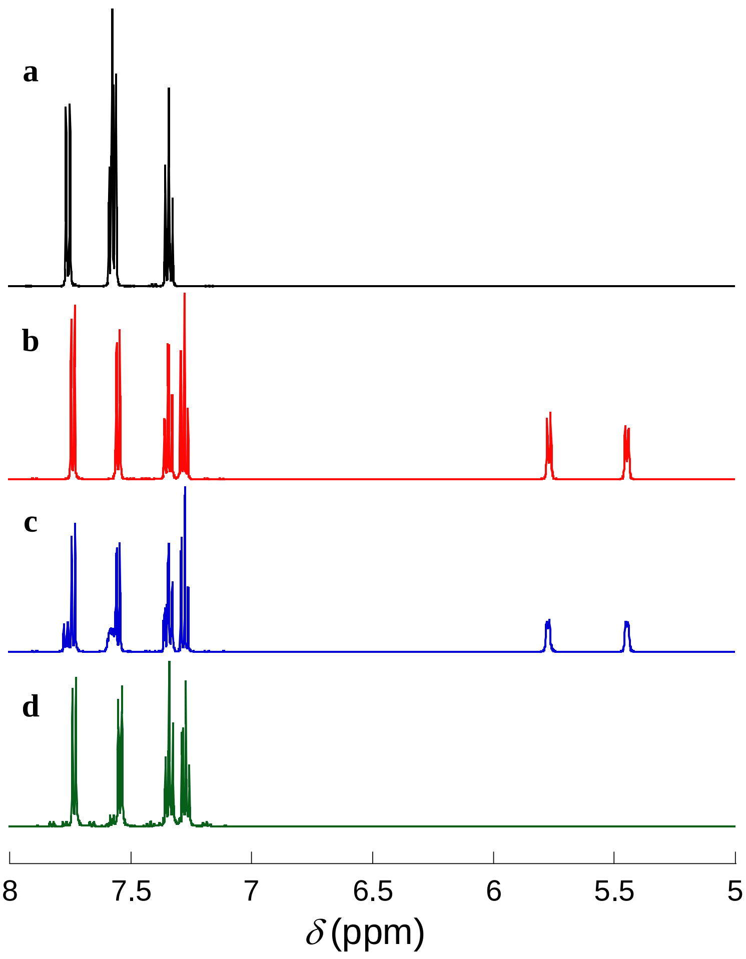

Supplementary Figure 4 | Electrolytic hydrogenation and deuteration of fluorenone. ^{1}H NMR spectra of fluorenone (**a**), fluorenol (**b**), the fluorenone after bulk electrolysis (**c**), and the fluorenone after deuteration (**d**). The bulk electrolysis was carried out by applying potential of -1.5 V (vs. Ag/AgCl) in a solution of 1 mM fluorenone and 0.1 M $(C_4H_9)_4NPF_6$ in water or AN/ D_2O (vol 5/1).

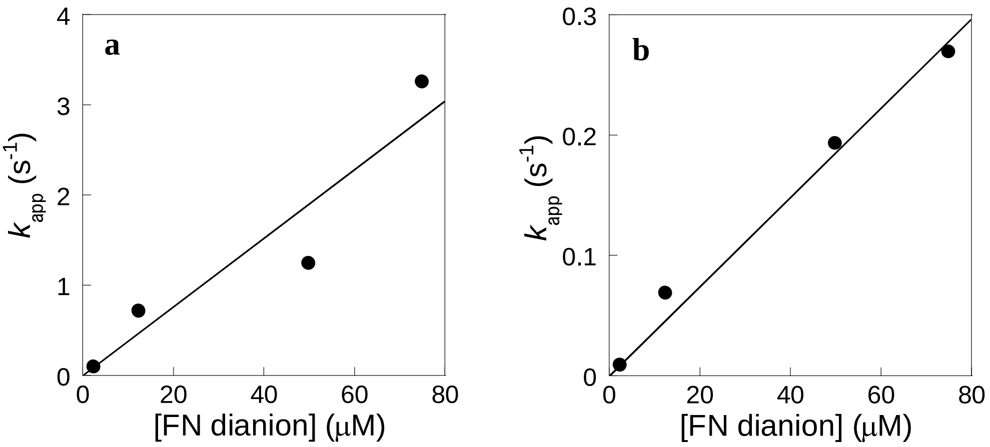

Supplementary Figure 5 | Rate constants of the proton-exchanging. k_{app} plots of hydroxyl (**a**) and cyclopentane (**b**) protons of fluorenol.

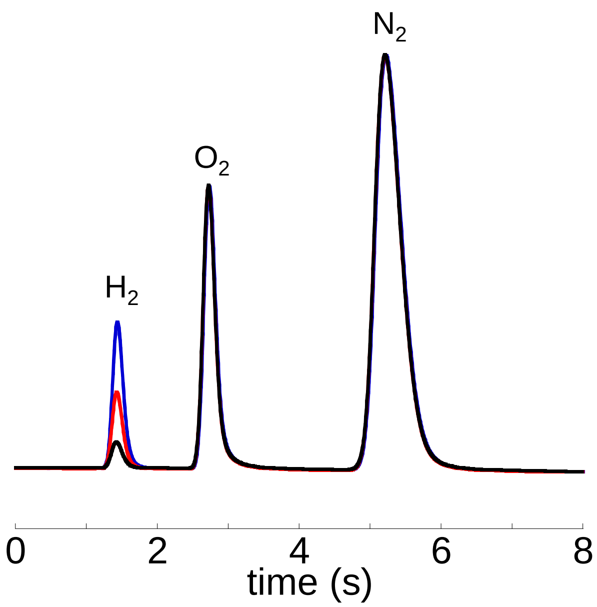

Supplementary Figure 6 | Gas chromatography analysis. Gas chromatography of the gas evolved from the fluorenol polymer after the dehydrogenation reaction for 10 min (black), 20 min (red), and 30 min (blue). The peak of H_2 increased with the reaction time, indicating the pure hydrogen gas evolution from the polymer.

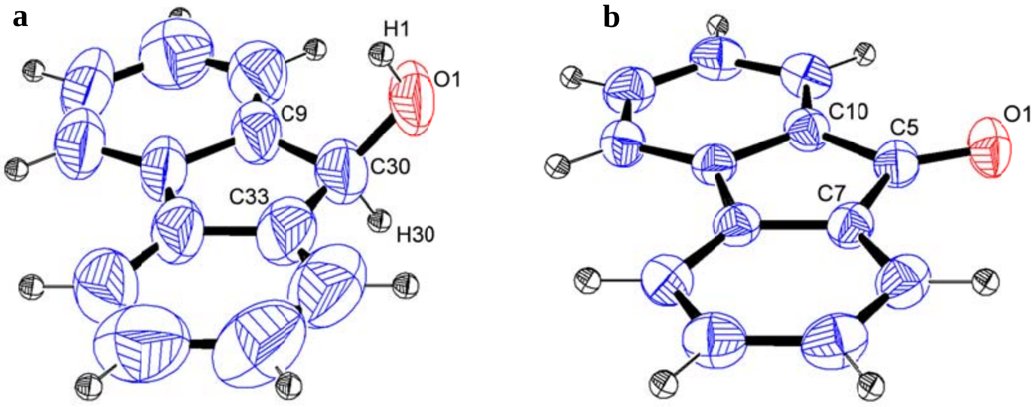

Supplementary Figure 7 | X-ray crystallographic structure of fluorenol and fluorenone (50% probability ellipsoids). a, ORTEP view of fluorenol. Selected bond lengths (Å) and angles (deg): O1–C30, 1.406(8); C30–C33, 1.516(11); C9–C33, 1.518(10); O1–H1, 0.840; C30–H30, 1.000; O1–C30–C9, 116.4(5); O1–C30–C33, 112.0(6); C30–O1–H1, 109.5; O1–C30–H30, 108.4. **b,** ORTEP view of fluorenone. Selected bond lengths (Å) and angles (deg): O1–C5, 1.223(15); C5–C10, 1.491(17); C5–C7, 1.490(17); O1–C5–C7, 127.5(12); O1–C5–C10, 126.9(12).

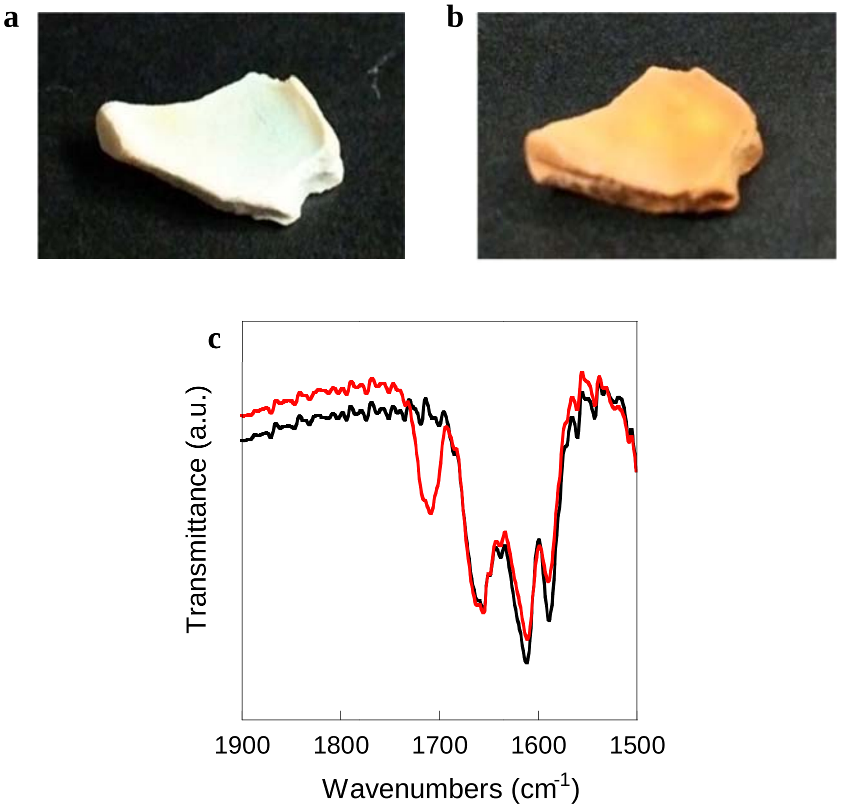

Supplementary Figure 8 | Analysis of the fluorenol polymer after the hydrogen evolution. Photograph of the fluorenol polymer specimen before (**a**) and after (**b**) the hydrogen evolution. **c,** IR spectrum of the fluorenol polymer before (black) and after (red) the hydrogen evolution.

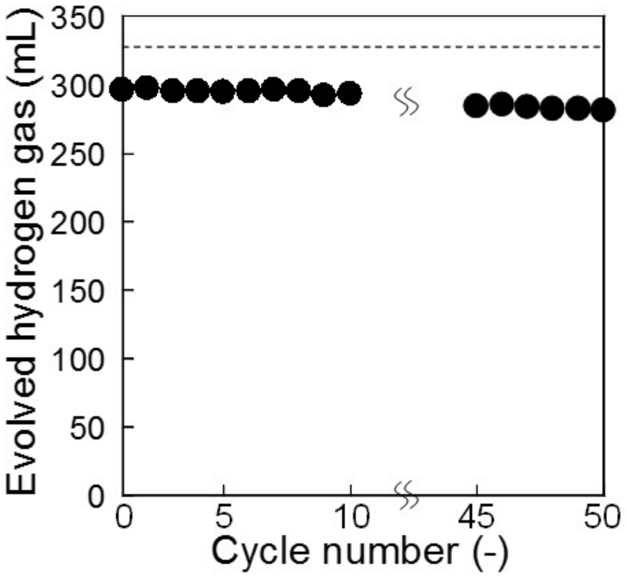

Supplementary Figure 9 | Cycle performance for hydrogen-fixing and -releasing in and from the composite sheet. Evolved hydrogen gas plots from the fluorenone/carbon composite sheet after electrolytic hydrogenation. Dashed line represents the theoretical or formula weight-based calculated hydrogen gas volume. The cycle performance was tested in twice, and each gas volume was within the error limits.

Supplementary Table 1 | Rate constants of the proton-exchanging reaction

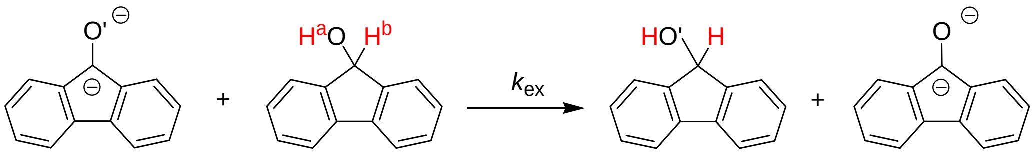

proton	k_{ex} ($M^{-1}s^{-1}$)
H^a	3.8×10^4
H^b	3.7×10^3

Supplementary Methods

2,7-Bis[2-(diethylamino)ethoxy]-9-fluorenone (17.0 g, 41.4 mmol) and sodium borohydride (11.3 g, 299 mmol) were dissolved and stirred in methyl alcohol (400 mL). Aqueous hydrochloric acid was added to neutralize the solution, evaporated, and the residue was extracted with chloroform. The organic layer was washed with brine, dried over anhydrous sodium sulfate, evaporated to remove the solvent, and dried: Color-less powder (2,7-bis[2-(diethylamino)ethoxy]-9-fluorenol, 15.8 g). ^{1}H NMR (500 MHz, $(CD_3)_2SO$): δ 7.51 (d, 2H), 7.10 (s, 2H), 6.89 (d, 2H), 5.78 (d, 1H), 5.37 (d, 1H), 4.03 (t, 4H), 2.78 (t, 4H), 2.55 (q, 8H), 0.98 (t, 12H); ^{13}C NMR (500 MHz, $(CD_3)_2SO$): δ 157.8, 148.3, 132.2, 119.8, 114.5, 111.4, 73.5, 66.6, 51.4, 47.0, 11.9; FAB-MS (m/z): $[M]^+$ calcd for $C_{25}H_{36}N_2O_3$, 412.6; found, 412.7; Analysis (calcd, found for $C_{25}H_{36}N_2O_3$): C (72.8, 72.8), H (8.8, 8.8), O (11.6, 11.7).

2,7-Bis[2-(diethylamino)ethoxy]-9-fluorenol (12.8 g, 31.1 mmol) and 1,3,5-tris(bromomethyl)benzene (7.39 g, 20.7 mmol) were dissolved in NMP (60 ml). The mixture was heated on a boat (10×21 cm^2) of Teflon™ at 80 °C for 5 h. The obtained polymer was washed with NMP, water, and methyl alcohol, and dried. Solid-state ^{13}C NMR spectrum of the obtained polymer gave the peaks (156.5, 147.5, 132.4, 118.3, 114.6, 111.8, 72.4 ppm) assigned to the fluorenol moiety, and the peaks

(46.9, 33.2 ppm) ascribed to the methylene carbons of the reactants were shifted.

A cyclic voltammogram of the fluorenone polymer-coated carbon in AN electrolyte (Supplementary Figure 3a) gave two quasi-reversible redox waves ascribed to the fluorenone unit in the polymer. On the other hand, the oxidation peaks disappeared in the CV in AN/water electrolyte. Reduction peaks decreased with multiple potential cycles and the polymer turned to a redox-inactive one, suggesting the formation of fluorenol units in the polymer.

Peaks assigned to the hydroxyl (5.48 ppm), cyclopentane (5.81 ppm), and phenyl (7.77, 7.59, 7.38, 7.31 ppm) protons of fluorenol appeared with the electrolytic reduction in AN/water (Supplementary Figure 4c). On the other hand, for the electrolytic reduction in AN/D_2O, only the peaks assigned to the phenyl protons (7.77, 7.58, 7.38, 7.31 ppm) of fluorenol appeared, which indicated 2D deuteration of the hydroxyl and cyclopentane protons of fluorenol (Supplementary Figure 4d).

The dianion of fluorenone was prepared by bulk electrolysis, by applying potential of -1.5 V (vs. Ag/AgCl) in the solution of 5 mM fluorenone and 0.1 M $(C_4H_9)_4NPF_6$ in AN- d_3. Mixtures of the dianion fluorenone and fluorenol (mol ratio 1/15–10000) were prepared in a N_2 glove box for NMR measurement. The rate constant of proton-exchanging (k_{ex}) was calculated by using equation $k_{app} = k_{ex}$ [FN dianion] and

$1/T^{obs} = 1/T + k_{app}$, where T is the relaxation time of each proton. Plots of k_{app} *vs*. [FN dianion] gave straight lines with the slopes of k_{ex} given in Table 1 (Supplementary Figure 5a and b).

The specimen of the fluorenol polymer was soaked in aqueous solution of the iridium catalyst (1.4 mol%) and warmed at 80 °C for 4 h. After hydrogen evolution, the color-less fluorenol polymer specimen turned reddish. A strong IR absorption peak ($\nu_{C=O}$ = 1710 cm^{-1}) supported that almost all of the fluorenol units in the polymer turned into the fluorenone units through hydrogen evolution, to yield the fluorenone polymer (Supplementary Figure 8).

To prepare the fluorenone polymer/carbon composite, 2,7-bis[2-(diethylamino)ethoxy]-9-fluorenone (9.50 g, 23.1 mmol) and 1,3,5-tris(bromomethyl)benzene (5.50 g, 15.4 mmol) were mixed with multi-wall carbon nanotube (6.43 g, Sigma-Aldrich) in NMP. The mixture was heated on a boat of Teflon™ at 80 °C for 5 h. The obtained fluorenone polymer/carbon composite sheet was washed with NMP, water, and methyl alcohol, and dried. The composite sheet was cut (14.2 g) and applied to the working electrode in the electrolytic hydrogenation.
